# Retrospective analysis of clinical and radiological outcomes of thoracolumbar vertebral fractures treated with monoplanar pedicle screws compared with fixed-axis and polyaxial screws

**DOI:** 10.3389/fmed.2025.1605583

**Published:** 2025-07-24

**Authors:** Wei Duan, Xin Zhao, Le Chang, Zhen Sun, Kangwei Lai, Jingchun Zhang, Buqi Tian, Zhengxu Ye

**Affiliations:** ^1^Department of Orthopedic, Xijing Hospital, Fourth Military Medical University, Xi’an, China; ^2^Department of Aerospace Medicine, Fourth Military Medical University, Xi’an, China; ^3^Department of Orthopedic, Xi’an Medical University, Xi’an, China

**Keywords:** thoracolumbar fracture, monoplanar pedicle screws, minimally invasive, radiological outcomes, biomechanical stability

## Abstract

**Background:**

Pedicle screw fixation is widely utilized in thoracolumbar fractures to restore vertebral height and spinal alignment. Screw head design varies among fixed-axis (rigid), polyaxial (multiplanar mobility), and monoplanar (coronal mobility) types, impacting surgical outcomes. This study compares clinical and radiological outcomes of these screws.

**Methods:**

Seventy-five patients (2020–2024) with thoracolumbar fractures were divided into three groups: Group A (fixed-axis screws, *n* = 31), Group B (polyaxial screws, *n* = 19), and Group C (monoplanar screws, *n* = 25). Operative time, blood loss, radiographic parameters (anterior body compression index, vertebral body angle, regional Cobb angle), and Visual Analogue Scale (VAS) scores were assessed preoperatively, postoperatively, and at 3 and 12 months.

**Results:**

Group C (monoplanar) demonstrated significantly lower blood loss (64 ± 11.1 mL vs. 308.6 ± 88.8 mL, *p* < 0.05) and shorter operative time (88 ± 8.2 min vs. 158.9 ± 27.8 min, *p* < 0.05) than Group A, with no significant differences compared to Group B. Postoperative VAS improved across all groups (*p* < 0.05), though Group A had slightly higher scores. Radiographic correction loss occurred in all groups at 12 months (*p* < 0.05), but was more pronounced in Group B. No complications (infection, nerve injury) were observed.

**Conclusion:**

Monoplanar pedicle screws, combining coronal mobility for minimally invasive placement and sagittal rigidity for stability, reduce blood loss and operative time compared to fixed-axis screws while maintaining comparable correction retention to polyaxial screws. These findings position monoplanar screws as a balanced option for thoracolumbar fracture fixation, optimizing minimally invasive benefits without compromising mechanical strength.

## 1 Introduction

Thoracolumbar spine fractures predominantly occur at the T12-L1 junction, a biomechanical transition zone characterized by increased segmental mobility between the rigid thoracic kyphosis and flexible lumbar lordosis (1, 2). This anatomical vulnerability frequently results in vertebral body collapse, sagittal plane deformity, and potential spinal canal compromise (3). Primary etiological factors include high-energy trauma (motor vehicle collisions, falls from height) and low-energy mechanisms in osteoporotic patients.

Surgical management of thoracolumbar fractures has evolved significantly since Hadra’s inaugural silver wire fixation system, with contemporary instrumentation emphasizing vertebral height restoration, spinal stability maintenance, neurological decompression, and early functional rehabilitation (4–6). Modern fixation systems employ three principal pedicle screw variants: fixed-axis, polyaxial, and monoplanar designs. While traditional open fixation with rigid fixed-axis screws established the historical standard, minimally invasive percutaneous techniques utilizing polyaxial screws have gained prominence due to demonstrated advantages in reduced surgical trauma, decreased blood loss, accelerated recovery, and lower complication rates (7–11). However, the enhanced sagittal-plane adaptability of polyaxial screws compromises construct stability and rotational control compared to fixed-axis systems.

The emerging monoplanar screw design represents an engineering compromise, permitting axial plane rotation for rod contouring while maintaining sagittal plane rigidity to preserve flexion-extension stability (12). This hybrid configuration has become our institutional preference for percutaneous fracture stabilization, though rigorous comparative evaluations of monoplanar systems remain scarce in the literature.

This retrospective cohort study conducts a comprehensive comparative analysis of monoplanar pedicle screw fixation versus conventional fixed-axis and polyaxial systems, evaluating both clinical outcomes and radiographic parameters in thoracolumbar fracture management.

## 2 Materials and methods

### 2.1 Patients materials

From 2020 to 2024, 75 patients (46 males and 29 females) aged from 17 to 67 years old of acute thoracolumbar fracture without neurological deficit were divided into three groups (Group A, Group B, and Group C), and were treated with pedicle fixation including fixed-axial, poly-axial and monoplanar pedicle screws, respectively. All patients involved in this study met the following inclusion criteria: (1) patients with single-level fracture between T11-L2 freed from disk, ligament instability or neurological injury; (2) absence of epidural sac compression or other severe injury; (3) TLICS (thoracolumbar injury classification severity and score) ranged from 4–6. The exclusion criteria were as follows: (1) patients with severe osteoporosis, defined as a bone mineral density (BMD) T-score ≤ − 2.5; (2) previously existing severe spine deformity, such as kyphosis and scoliosis; (3) combined with other sites of fracture. Group allocation was not randomized. Patients were assigned to each group based on the time period of treatment and surgeon preference, which may introduce selection bias. However, demographic characteristics and injury severity (TLICS score) were comparable among groups, minimizing potential confounding factors. The follow-up period ranged from 11 to 13 months, with a mean duration of 12 months. We access the clinical outcomes of all the patients by comparing Visual Analogue Scale (VAS) preoperatively, immediately after operation and three months after operation and twelve months after operation.

### 2.2 Operation methods

**Figure 1:**
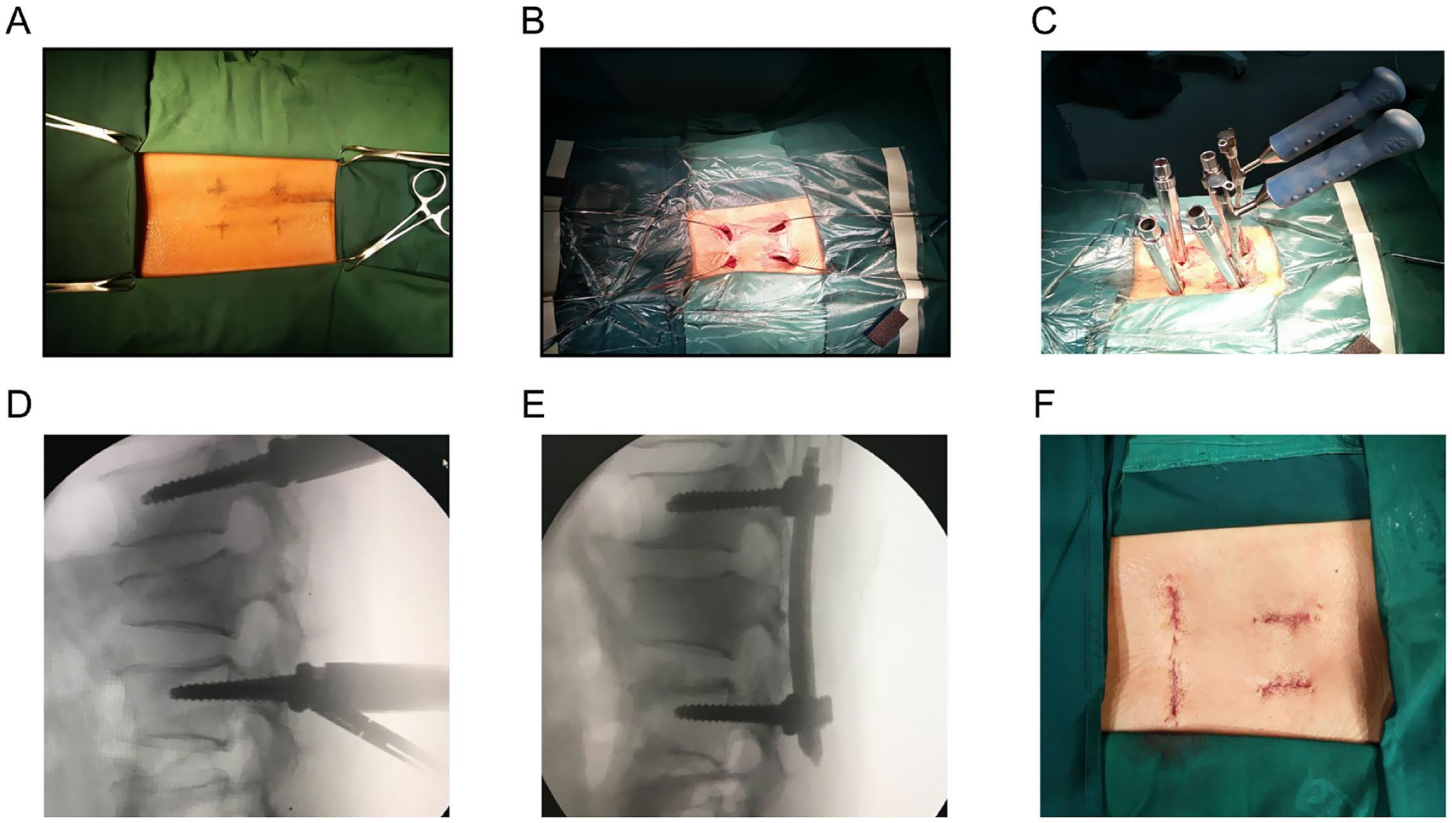
**(A)** Pedicle access of minimally invasive percutaneous pedicle screws fixation using monoplanar screws. **(B)** Guidance wires were placed. **(C)** Rod-screw fixation system was installed. **(D)** Intraoperative lateral fluoroscopy was taken to confirm the fixation before distraction. **(E)** Intraoperative lateral fluoroscopy was taken to evaluate the reduction and restoration after appropriate distraction and compression. **(F)** Four incisions were closed (each 10–15 mm).

Under general anesthesia with endotracheal intubation, all surgeries were performed by the same lead spine surgeon, assisted by a consistent team of experienced spine surgeons, to ensure uniform surgical technique and reduce operator-related variability. The surgical team was led by a chief surgeon who had independently performed more than 50 similar thoracolumbar fracture fixation procedures before the initiation of this study, thereby minimizing potential biases related to the learning curve. All patients were placed on a radiolucent operating table in the prone position with the thorax, pelvis and both ankles supported by the rudder cushions to make the spine in a hyperextension position (Figure 1).

#### 2.2.1 Group A

Preoperative location of the involved region including the upper and lower adjacent vertebra was accomplished under the guidance of the posteroanterior fluoroscopic images and then marked. A standard mid-line incision, appropriately 10 cm, was made centered over the fractured region; longitudinally incised the supraspinous ligament; blunt detached the paraspinal muscles till the bilateral laminas and facet joints were exposed. The fix-axial pedicle screws and rod (made by Johnson & Johnson, Inc.) were installed routinely without fusion or laminectomy; to obtain correction of the traumatic deformity and normal height of the vertebral body, rod-screw system should be fastened properly; precise location of the screws and deformity correction should be confirmed by the posteroanterior and lateral radiological images.

#### 2.2.2 Group B

On the basis of the posteroanterior fluoroscopic images, the fractured vertebra body and the adjacent upper and lower one’s pedicle should be ensured, and the pedicle access were marked on the surface of the skin. Totally six proper incision, appropriately 10-15 mm, were made seriatim along the marked line and extended into the subcutaneous tissue. Positioning needles were placed at the pedicle entry points under intraoperative X-ray fluoroscopic guidance (posteroanterior and lateral views). After confirming the correct trajectory via fluoroscopy, guidewires were inserted along the needles, which were then removed. Every pedicle pilot hole was tapped and then suitable poly-axial pedicle screws (made by Medtronic, Inc.) were inserted through the guidance wires; the guidance wires were removed carefully after accurate position of the screw. According to the curvature of the spine, proper rods were pre-bent and sited along the percutaneous pedicle screws. To obtain enough reduction of the fractured vertebra body, appropriate distraction or compression as well as slight lordosis were conducted over the screws-rod system. Posteroanterior and lateral radiograph was obtained to determine satisfactory fixation and reduction.

#### 2.2.3 Group C

The operative procedure in Group C was largely similar to that of Group B, except for the type of screws used. However, a key distinction is that no pedicle screws were inserted into the fractured vertebral body in Group C; only the adjacent upper and lower vertebrae were instrumented. This fracture-level-sparing technique was intentionally applied to reduce implant cost and preserve segmental mobility. Intraoperative blood loss and operation time were recorded according to the operative documents. Blood loss was calculated by summing the volume collected in the suction canister (after deducting irrigation fluid) and the estimated amount absorbed by surgical sponges, calculated by weighing sponges before and after use. After operation, all the patients were routinely accepted antibiotics proactively and early ambulation was encouraged 24 h after the surgery. Specifically, all patients were administered intravenous cefuroxime 1.5 g every 8 h for 24 h postoperatively to minimize the risk of infection. This regimen was applied consistently across all three groups to ensure standardization of infection prevention measures. All patients followed a standardized postoperative rehabilitation protocol. A thoracolumbosacral orthosis (TLSO) brace was prescribed and worn during ambulation for 6 weeks. Early mobilization was encouraged, with gradual weight-bearing as tolerated starting 24 h postoperatively. Additionally, all patients were instructed to wear a thoracolumbosacral orthosis (TLSO) brace for 6 weeks after surgery during ambulation, following a standardized rehabilitation protocol across all groups. Twelve months later, pedicle screws could be removed if the patients had satisfactory recovery in terms of clinical and radiological outcomes.

### 2.3 Radiographic evaluation

**Figure 2:**
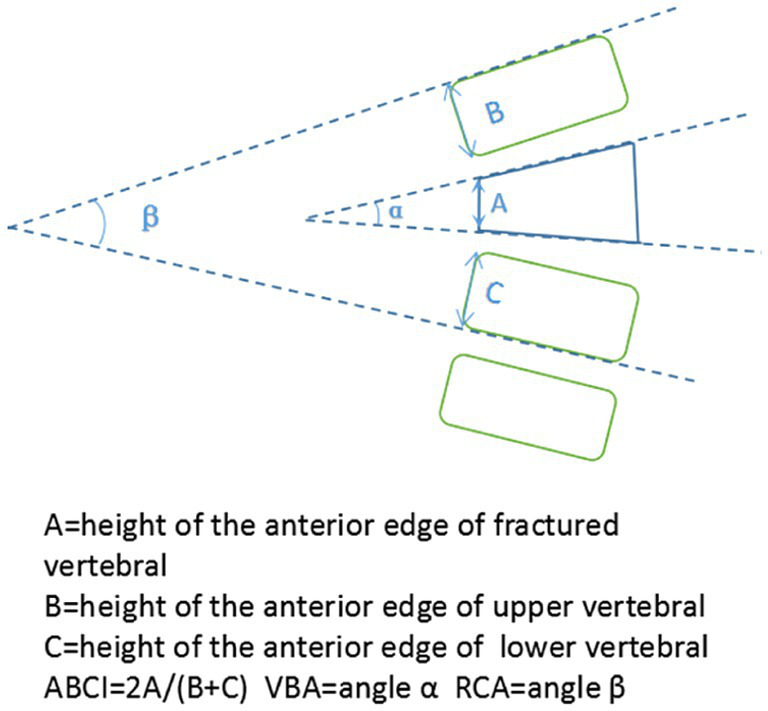
Imaging parameters used in this study.

Plain films were obtained preoperatively, immediately after surgery, 3 and 12 months after operation. Radiographic parameters consisted of ABCI (anterior body compression index), VBA (vertebral body angle), RCA (regional cobb angle) (Figure 2). The ABCI and VBA was applied to reflect the restoration of the fractured vertebra body. The RCA was defined by the angle between the superior endplate of the upper vertebral and the inferior endplate of the lower vertebra, which was used to assess the correction changes correlative with the pedicle screws inserted. All imaging evaluations were performed by a single independent orthopedic surgeon who was not involved in the surgical procedures. The evaluator was blinded to group allocation to reduce potential subjective bias.

### 2.4 Clinical evaluation

The VAS scores, which is measured for the assessment of the back pain, was obtained preoperatively, immediately after operation, 3 and 12 months after operation.

### 2.5 Statistical analysis

Demographic, radiological and clinical outcomes of the Group C were compared horizontally with Group A and Group B respectively, and longitudinally compared with itself at variable times including preoperative, immediately after operation, 3 months and 1 year after operation. Difference of measurement data was compared with analysis with univariate ANOVA. Comparison of categorical variables was taken using the chi-square test. All *p* values<0.05 were considered statistically significant. The SPSS software program for windows V19.0 was used to analyze all the data.

## 3 Results

**Figure 3:**
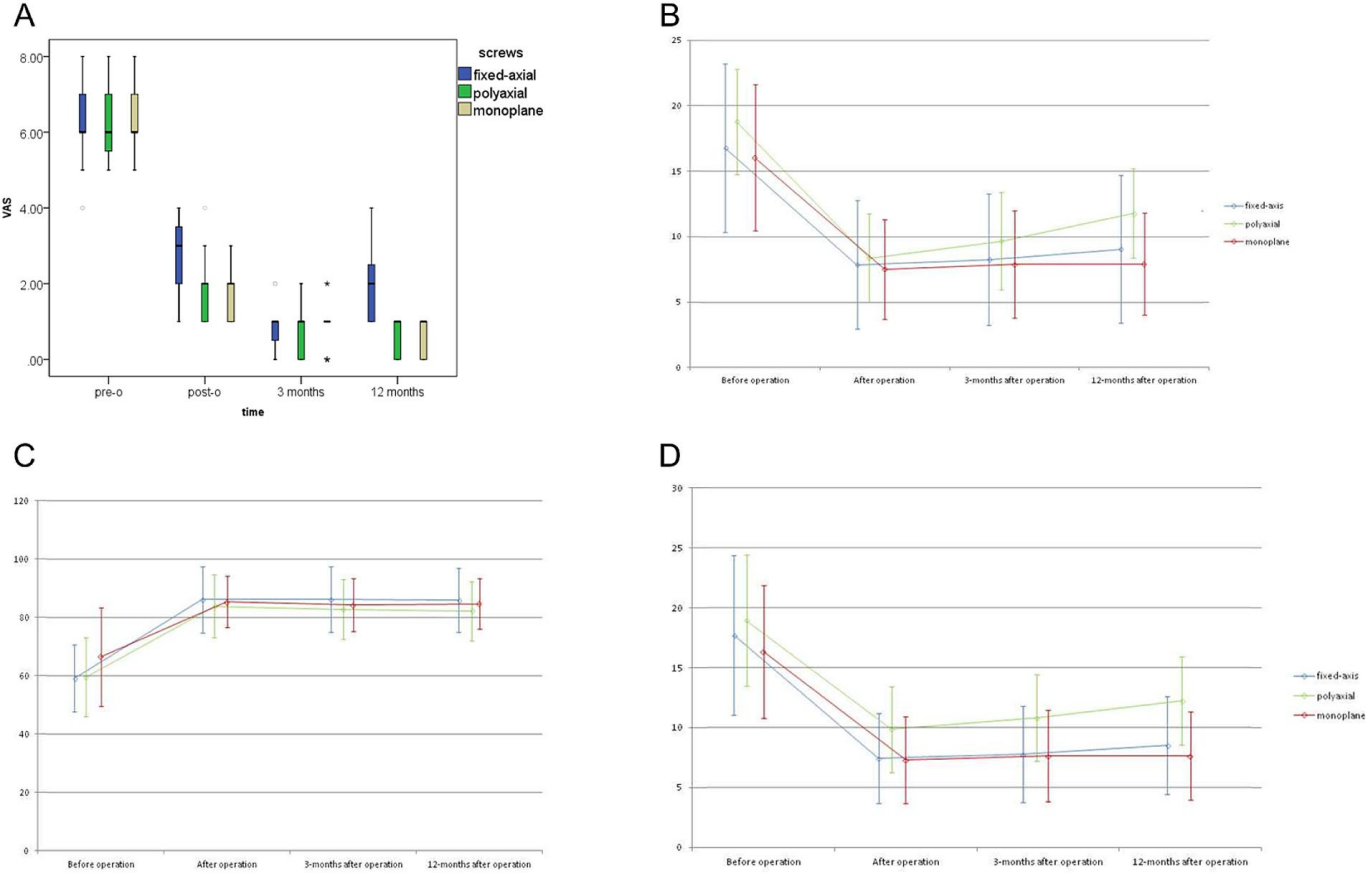
**(A)** VAS scores of different groups at the same time during the entire study period. **(B)** Radiological parameters at different time points of Group A. **(C)** Radiological parameters at different time points of Group B. **(D)** Radiological parameters at different time points of Group C.

Clinical outcomes. The average blood loss and operation time of Group C was 64 ± 11.1 mL,88 ± 8.2 min, which were obviously less than Group A (308.6 ± 88.8 mL, 158.9 ± 27.8 min) (*p* < 0.05) and had no significant difference compared with Group B (69.7 ± 12.4 mL, 92.6 ± 10.6 min) (*p* > 0.05) (Table 1). No significant difference was observed when the preoperative VAS of three different groups were compared. Postoperatively (almost 1st day after operation), the VAS of three groups all were significantly lower than those before operation (*p* < 0.05), additionally, there was no significant difference when compared with each other (*p* > 0.05) at the same period of time (Figure 3). However, VAS of Group A was slightly higher than those of the other two groups especially after operation immediately.

**Table 1:** Clinical outcomes of different groups.

Group/parameter	Blood loss (ml)	Operation time (min)
Group A	308.6 ± 88.8	158.9 ± 27.8
Group B	69.7 ± 12.4	92.6 ± 10.6
Group C	64 ± 11.1	88 ± 8.2

Radiological outcomes. The RCA, VBA and ABCI in all groups decreased significantly immediately after fixation operation, and maintained well until the last visit (Tables 2–5). Furthermore, all the parameters during the postoperative course showed a shifty tendency effect of time which was more pronounced in Group B compared with Group A and Group B (Figure 3). But this change was not found to be statistically significant in comparison with those after operation immediately.

**Table 2:** Radiological parameters in different time of Group A.

Time/parameter	RCA	VBA	ABCI (%)
Before operation	16.77 ± 6.43	17.71 ± 6.64	59.2 ± 11.5
After operation	7.87 ± 4.92	7.42 ± 3.78	86.2 ± 11.4
3-months after operation	8.26 ± 5.03	7.74 ± 4.01	86.3 ± 11.1
12-months after operation	9.06 ± 5.62	8.48 ± 4.10	86.0 ± 11.0

**Table 3:** Radiological parameters in different time of Group B.

Time/parameter	RCA	VBA	ABCI (%)
Before operation	18.79 ± 4.05	18.95 ± 5.46	59.6 ± 13.6
After operation	8.37 ± 3.39	9.84 ± 3.59	83.9 ± 10.8
3-months after operation	9.68 ± 3.70	10.79 ± 3.61	82.9 ± 10.3
12-months after operation	11.79 ± 3.41	12.21 ± 3.691	82.3 ± 10.2

**Table 4:** Radiological parameters in different time of Group C.

Time/parameter	RCA	VBA	ABCI (%)
Before operation	16.04 ± 5.59	16.32 ± 5.57	66.6 ± 16.9
After operation	7.52 ± 3.81	7.28 ± 3.62	85.5 ± 8.9
3-months after operation	7.88 ± 4.07	7.64 ± 3.82	84.4 ± 9.1
12-months after operation	7.92 ± 3.88	7.64 ± 3.70	84.8 ± 8.6

**Table 5:** *P* value of comparison between Group C with Group A and B about the RCA/VBA/ABCI at different time.

Time	Group	Group	p(RCA)	p(VBA)	p(ABCI)
Pre-operation	C	A	0.629	0.393	0.055
		B	0.113	0.156	0.104
Post-operation	C	A	0.758	0.888	0.822
		B	0.511	<0.05*	0.61
3-months after operation	C	A	0.184	0.922	0.493
		B	0.751	<0.05*	0.617
12 months after operation	C	A	0.357	0.42	0.65
		B	<0.05*	<0.05*	0.417

When we assess RCA, reduction was statistically significant in all groups immediately after fixation operation, and no significant difference was found between each other. However, 12 months after operation, there was significant difference between Group B (11.79 ± 3.41) and Group C (7.92 ± 3.88). Either after operation immediately or 3,12 months after operation, the VBA in Group C (7.28 ± 3.62, 7.64 ± 3.82, 7.64 ± 3.70) was significantly (*p* < 0.05) higher than Group B (9.84 ± 3.59,10.79 ± 3.61, 12.21 ± 3.691). No statistically significant difference was observed between Group A and Group C at the same period of time (Table 5).

Complications. No major complications such as instrumentation failure, infection, bedsores, or neurological injury were observed. In addition, patients were routinely monitored for minor postoperative events, including deep vein thrombosis (DVT), brace-related discomfort, and delayed wound healing, none of which were identified during the follow-up period. However, something interesting should not be ignored that the thinner patients preferred to feel more uncomfortable after operation immediately in horizontal position potentially resulting from the fixation.

## 4 Discussion

The optimal management strategy for thoracolumbar burst fractures—surgical intervention versus conservative treatment—remains debated in clinical practice (13). While non-operative approaches may suffice for neurologically intact patients with stable fractures, surgical modalities (open or minimally invasive) offer distinct advantages including immediate deformity correction, spinal canal decompression, and accelerated pain relief (14). Treatment decisions should be guided by validated classification systems such as the AO Spine criteria or TLICS (Thoracolumbar Injury Classification and Severity Score), which stratify injury patterns and neurological status to inform therapeutic pathways (15–17). In this study, we employed TLICS (scores 4–6) as the inclusion criterion to standardize surgical indications across cohorts. All 75 patients completed the scheduled follow-up assessments at 3 and 12 months. No cases were lost to follow-up, and all were included in the final analysis.

Contemporary surgical techniques broadly categorize into traditional open procedures and minimally invasive surgery (MIS). MIS demonstrates well-documented benefits over open approaches, including reduced intraoperative blood loss, diminished postoperative pain, lower infection rates, and preservation of paraspinal musculature (18–20). However, MIS applicability remains constrained in cases requiring extensive decompression or involving complex posterior ligamentous injuries.

Group B has gained popularity due to its minimally traumatic profile and cost-effectiveness. Nevertheless, biomechanical limitations associated with polyaxial designs-specifically residual kyphotic forces and sagittal plane instability-may predispose to progressive correction loss and vertebral height collapse. Studies have suggested that fracture-level inclusion in short-segment constructs or long-segment instrumentation enhances deformity correction durability (21). Notably, Basaran et al. demonstrated that fracture-level-augmented short-segment fixation preserves spinal mobility better than long-segment alternatives (22), rationalizing our surgical protocol for Group B.

Pedicle screw evolution has paralleled MIS advancements since their inaugural percutaneous application in 1994 (23, 24). While fixed-axis screws remain incompatible with percutaneous rod insertion due to restricted trajectory adjustability, polyaxial designs (Group B) resolve this limitation through multiplanar screw-head articulation. However, our prior biomechanical analysis revealed that polyaxial systems exhibit significantly reduced static/dynamic construct stiffness compared to fixed-axis counterparts, potentially compromising deformity correction. Group C represent a biomechanical hybrid, permitting axial rotation for rod engagement while maintaining sagittal plane rigidity to mitigate correction loss—a feature corroborated by equivalent stiffness to fixed-axis systems in laboratory testing.

This retrospective analysis compared three surgical cohorts: Group A underwent traditional open fixation with fixed-axis screws without decompression; Group B received percutaneous fracture-level-augmented fixation using polyaxial screws; Group C was treated with percutaneous non-fracture-level fixation employing monoplanar screws. Demographic parameters (age, gender, fracture level, TLICS scores) showed intergroup homogeneity. Operative metrics revealed significantly greater blood loss (308.6 ± 88.8 mL vs. 69.7 ± 12.4 mL/64 ± 11.1 mL) and longer duration (158.9 ± 27.8 min vs. 92.6 ± 10.6 min/88 ± 8.2 min) in Group A versus Groups B/C (*p* < 0.01). Although all groups achieved significant postoperative pain reduction (VAS), Group A demonstrated marginally higher residual pain scores at immediate postoperative (2.7 ± 1.0 vs. 1.7 ± 0.9/1.6 ± 0.6) and post-implant removal (1.9 ± 1.0 vs. 0.7 ± 0.5/0.7 ± 0.5) intervals, likely attributable to open approach-related soft tissue trauma. In addition to the three systems discussed, hybrid fixation strategies—such as constructs combining monoaxial and polyaxial screws—have been explored in the literature to balance rigidity and rod insertion flexibility. Some studies suggest that such combinations may provide improved biomechanical performance in certain fracture patterns. However, these techniques come with limitations, including increased procedural complexity, cost, and potential cement-related complications. Compared to these alternatives, the monoplanar system in our study offered a simpler, cost-effective option that maintained sagittal plane stability and surgical efficiency in non-osteoporotic patients.

Radiologically, all groups maintained vertebral body angle (VBA) and regional Cobb angle (RCA) corrections throughout follow-up, with no significant intergroup differences between Groups A and C. Notably, Group C exhibited less correction loss in VBA/RCA than Group B (*p* = 0.08), potentially explained by differential screw-rod interface mechanics. Fixed-axis screws achieve rigid screw-rod coupling via U-shaped groove engagement, whereas polyaxial/monoplanar designs permit subtle micromotion despite final tightening—a phenomenon exacerbated by intraoperative derotation maneuvers. The monoplanar system’s sagittal plane constraint likely mitigates screw-head swing, enhancing construct stability relative to polyaxial counterparts. Additionally, Group C’s non-fracture-level fixation reduced implant costs versus Group B.

However, this study still has some limitations. The relatively short follow-up period (11–13 months) may have limited our ability to detect long-term complications such as delayed correction loss, instrumentation failure, or late-onset adjacent segment disease. Additionally, the relatively small sample size may reduce the statistical power to identify subtle intergroup differences, particularly in rare complication rates. Therefore, our findings should be interpreted with caution and further validated by larger-scale, long-term studies. Besides, Economic constraints precluded implant removal in 38% of patients, limiting assessment of post-explant correction loss.

## 5 Conclusion

This comparative analysis demonstrates that monoplanar pedicle screw systems harmonize the advantages of MIS (reduced blood loss, shorter operative duration) with biomechanical stability comparable to traditional open fixation. Percutaneous monoplanar fixation achieved equivalent radiographic outcomes to open fixed-axis systems while demonstrating superior maintenance of sagittal correction compared to polyaxial constructs. Clinically, monoplanar designs provided pain relief comparable to polyaxial systems while reducing implant-related costs. The sagittal plane constraint inherent to monoplanar screws appears critical for preventing correction loss, validating their biomechanical hybrid design. These results position monoplanar pedicle screws as a viable MIS alternative for thoracolumbar fracture stabilization, particularly when fracture-level augmentation is clinically contraindicated. Future investigations should address long-term outcomes and post-explant biomechanical behavior through extended follow-up and larger multicenter cohorts.

## Data Availability

The raw data supporting the conclusions of this article will be made available by the authors, without undue reservation.
